# IsoformEx: isoform level gene expression estimation using weighted non-negative least squares from mRNA-Seq data

**DOI:** 10.1186/1471-2105-12-305

**Published:** 2011-07-27

**Authors:** Hyunsoo Kim, Yingtao Bi, Sharmistha Pal, Ravi Gupta, Ramana V Davuluri

**Affiliations:** 1 Center for Systems and Computational Biology, The Wistar Institute, 3601 Spruce Street, Philadelphia, PA 19104-4268, USA

## Abstract

**Background:**

mRNA-Seq technology has revolutionized the field of transcriptomics for identification and quantification of gene transcripts not only at gene level but also at isoform level. Estimating the expression levels of transcript isoforms from mRNA-Seq data is a challenging problem due to the presence of constitutive exons.

**Results:**

We propose a novel algorithm (IsoformEx) that employs weighted non-negative least squares estimation method to estimate the expression levels of transcript isoforms. Validations based on *in silico *simulation of mRNA-Seq and qRT-PCR experiments with real mRNA-Seq data showed that IsoformEx could accurately estimate transcript expression levels. In comparisons with published methods, the transcript expression levels estimated by IsoformEx showed higher correlation with known transcript expression levels from simulated mRNA-Seq data, and higher agreement with qRT-PCR measurements of specific transcripts for real mRNA-Seq data.

**Conclusions:**

IsoformEx is a fast and accurate algorithm to estimate transcript expression levels and gene expression levels, which takes into account short exons and alternative exons with a weighting scheme. The software is available at http://bioinformatics.wistar.upenn.edu/isoformex.

## Background

The central dogma of molecular biology "the flow of genomic information from DNA to RNA to protein" in multi-cellular organisms is much more complex than originally thought [1]. An important aspect of this complexity is the generation of multiple transcript isoforms from a single gene in a genomic locus, due to the use of alternative initiation and/or termination of transcription and alternative splicing of pre-mRNAs [2-4]. Although the functional consequence of differential expression of alternative isoforms for some genes has been known, the advent of massive parallel sequencing technology has facilitated the study of transcript isoforms at genome-scale. In fact, recent evidence suggests that almost all multi-exon human genes have more than one mRNA isoform [5,6]. For example, the deep sequencing of cDNA fragments of 15 human tissue and cell line transcriptomes showed that 92-94% of human genes undergo alternative splicing [5]. The transcript variants are differentially expressed across different tissue/cell types, developmental stages and disease conditions. Moreover, for many genes, different transcript isoforms can lead to different protein isoforms with distinct functions. Therefore, in order to study the gene function at isoform level, it is necessary to know the expression of each transcript in various physiological and disease conditions.

With the advent of next-generation sequencing (NGS) technologies and decreasing cost per base, mRNA-Seq approach has become a desirable method to get a complete view of the transcriptome and to detect rare transcripts and isoforms [4-10]. mRNA-Seq experiments generate millions of short sequence reads that are sequenced from expressed transcripts. The majority of these short sequence reads can be mapped to exon regions of the genome and exon-exon junction regions of a transcriptome. Therefore, mRNA-Seq has been used for splice junction identification and alternative splicing detection [11-15], and for novel transcript identification through transcript assembly [16-18]. The assay provides sensitive and accurate digital counts for the exon regions of expressed transcripts in a given sample. The count of short sequence reads for each exon region is the sum of counts belonging to the exon region of different transcript isoforms that are expressed in the sample. Therefore, estimating the transcript-level expression from the collection of counts of short read sequences that map to exons or exon slices is a computational challenging problem, which has been recently attempted by some programs such as rSeq [19] based on maximum likelihood estimation, Cufflinks [20] extended from rSeq, and RSEM [21] considering sequencing mapping uncertainty. Bayesian Analysis of Splicing IsoformS (BASIS) [22] is another recent method that computes transcript levels from coverage of known exons by taking advantage of isoform-specific nucleotide positions from each transcript isoform. BASIS focuses on detecting the differentially expressed transcript isoforms between two conditions and cannot infer the expression levels under each condition. GPSeq using a two-parameter generalized Poisson (GP) model to the position-level read counts can estimate gene/exon expression and identify differentially expressed genes/exons [23]. However, GPSeq was not designed for estimating transcript expression levels. Another recent method [24] applied a linear model to estimate the ratios of known isoforms in a sample by incorporating the non-uniformity of mRNA-Seq read positions along the targeted transcripts as a key feature into its algorithm. However, the software is not publicly available for use.

Here, we present a novel method, called IsoformEx, which is based on weighted non-negative least squares. We compared IsoformEx with some of the published methods in accurately estimating the expression levels of transcript isoforms. Since Cufflinks has additional function of transcript assembly to identify novel transcripts, we only used its function of transcript level estimation extended from rSeq algorithm for the comparison studies. The publicly available software to estimate mRNA-Seq transcript expression with read mapping uncertainty (RSEM) was also used in the comparison studies. For fair comparisons, we used both simulated mRNA-Seq data and published real mRNA-Seq data [5] obtained from breast normal (HME) and cancer (MCF-7) cell lines.

## Results

### Basic logic of IsoformEx

**Figure 1:**
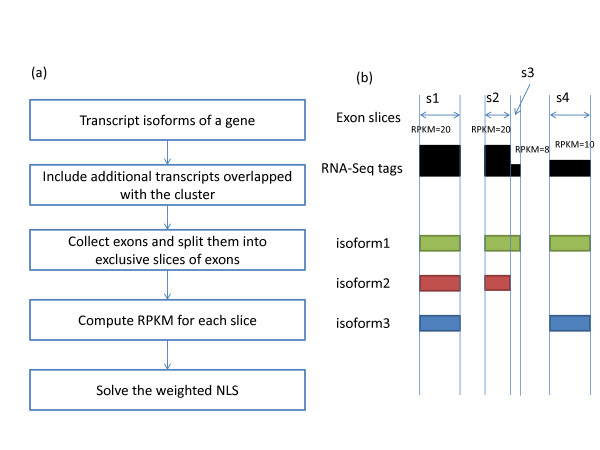
**Algorithm and exon slices**. (a) Algorithm flow chart. (b) The exon slices were determined by the genomic structures of the transcript isoforms having overlapping exons. The lower weight was applied for the shorter exon slice. This example shows four exon slices (s1-s4) obtained from three transcript isoforms. The observed RPKM of the smallest exon slice (s3) was smaller than 10 because its length is small.

**Figure 2:**
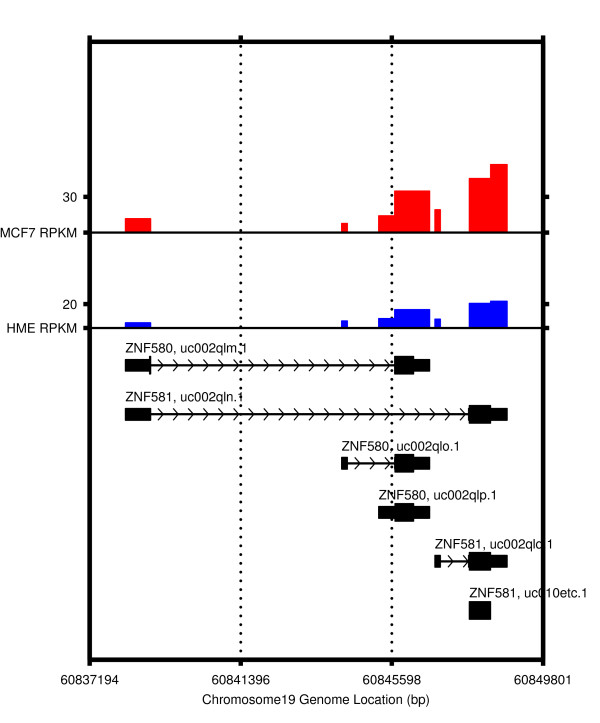
**RPKM values of transcript isoforms of *ZNF580 *and *ZNF581***. RPKM values of transcript isoforms of *ZNF580 *and *ZNF581 *in the breast normal cell line (HME) and cancer cell line (MCF-7). The total number of exon slices in this transcript block having six overlapping transcripts was eight, and approximated RPKM values of the exon slices in MCF-7 cell line were α(s1) ≈ 10, α(s2) ≈ 8, α(s3) ≈ 14, α(s4) ≈ 30, α(s5) ≈ 20, α(s6) ≈ 0, α(s7) ≈ 45, and α(s8) ≈ 60, where α(·) is the RPKM value of an exon slice (for example, α(s1) and α(s8) are the RPKM values of the first and the eighth exon slices). Although the sixth exon slice can be expressed from uc002qln.1 and uc002qlq.1, the observed value was close to 0 due to its very small exon slice size. In order to handle this small exon effect (observed RPKM of very small exon slices is usually not reliable), we applied a lower weight.

IsoformEx generates expression estimates both at gene and transcript levels from mapped mRNA-Seq reads (see Figure 1 for flowchart of IsoformEx algorithm and an example of gene with 3 transcript variants). We illustrate the basic logic of IsoformEx by using the sequence reads (from MCF-7 cell line [5]) that map to a genomic locus with two overlapping genes (*ZNF580 *and *ZNF581*) (see Figure 2). Let us denote RPKM of each transcript as *θ*(transcriptID) and RPKM of the exon slice as α(sliceID) in Figure 2. A transcript block is defined as a set of transcripts overlapped in a genomic locus. We identify the exon slices that are specific to each transcript in a transcript block, and call those exon slices as 'discriminative exon slices' within a transcript block. The RPKM values of the discriminative exon slices are used as a major factor for estimating the expression levels of corresponding transcripts having the discriminative exon slices. The total number of exon slices in this transcript block with six overlapping transcripts is eight, and the approximated RPKM values of the exon slices in MCF-7 cell line are α(s1) ≈ 10, α(s2) ≈ 8, α(s3) ≈ 14, α(s4) ≈ 30, α(s5) ≈ 20, α(s6) ≈ 0, α(s7) ≈ 45, and α(s8) ≈ 60, where s1, s2, ..., and s8 are the first, the second, ... , and the eighth exon slices. The highest expressed last exon slice is common to the two transcripts (uc002qln.1 and uc002qlq.1). The basic logic of estimation is as follows (see Figure 2, Additional File 1 Tables S1-S2 and Figure S1 for more detailed information). Since RPKM of the discriminative exon slice of uc002qlq.1 transcript is α(s5) ≈ 20, we expect *θ*(uc002qlq.1) ≈ 20 and *θ *(uc002qln.1) ≈ 40 by using α(s8) ≈ 60. However, RPKM of the first slice is only α(s1) ≈ 10. Thus, *θ*(uc002qln.1) may be lower than 40 (let us say *θ*(uc002qln.1) ≈ 35), and *θ*(uc002qlm.1) may be very low or zero (let us say *θ*(uc002qlm.1) ≈ 0). The RPKM of the discriminative exon slice of uc002qlo.1 is α(s2) ≈ 8. And, the RPKM of the discriminative exon slice of uc002qlp.1 is α(s3) ≈ 14. Note that the seventh exon slice is common to three transcripts (uc002qln.1, uc002qlq.1, and uc010etc.1). Since *θ*(uc002qln.1) ≈ 35 and *θ*(uc002qlq.1) ≈ 20, we expect that α(s7) is higher than 55. However, actual α(s7) is only about 45. Thus, *θ*(uc010etc.1) can be close to zero. As illustrated in this example, the discriminative exon slices and the concept of non-negativity are important for estimating transcript expression levels. The discriminative exon slices are highly weighted in the weighted non-negative least squares computation. Since the RPKM value of very small exon slice (s6) is not reliable for estimation (*i.e*. small exon effect), the small exon slice is lowly weighted. Note that the observed RPKM values and estimated transcript expression levels should be non-negative.

### Performance comparison using mRNA-Seq simulated data

Since the true expression levels of all transcript isoforms are unknown, we first designed an mRNA-Seq simulator to obtain artificial short sequence reads after assigning transcript expression levels. In order to mimic the distribution of transcript expression levels, we fitted an exponential decay function to the real mRNA-Seq data obtained from MCF-7 cell line, and used the fitted distribution to randomly assign expression levels to all transcripts. The assigned expression levels were therefore treated as known true expression levels in the simulated mRNA-Seq data for performance comparisons. In the mRNA-Seq simulator, the number of fragments for each transcript was determined and the transcript was randomly fragmented. The first 32 bp (*i.e*. tag length) of the fragment sequence and the first 32 bp of the reverse complementary sequence of the fragment were stored. Single nucleotide substitutions were introduced in the sequences, which reflect the 0.1% rate of SNPs in the human genome. In addition, nucleotide substitutions were introduced to account for current rate of sequencing errors (~ 1%). The sequencing error rate at each location of a sequence read of length 32 bp was obtained from mismatch information of tag mapping and Phred sequence quality scores in the MCF-7 data [5]. The sequencing error rates were fitted to an exponential function and the fitted function was used for introducing sequencing errors for each sequencing cycle. The sequencing error ratios at 1-25th nucleotides were much lower than those at 26th to 32nd nucleotides. The error ratio at 26th position was about 1% of all reads. In order to simulate sampling of fragments, we randomly selected 10% of reads and stored them to a FASTQ file. About 93% of the simulated sequence reads were then mapped to the human genome.

To assess the performance of IsoformEx, we compared it with Cufflinks (v0.9.3) since it incorporated an estimation algorithm of rSeq and the latest information about mRNA-Seq protocol. The mRNA-Seq reads were aligned to hg18 version of the human genome downloaded from the UCSC genome browser database [25]. The splice junction library was built from the UCSC transcriptome and sequence alignment was performed using Bowtie (v0.12.3) [26]. The transcripts information was converted to GTF file format and Cufflinks used the transcript information through the -G/--GTF option to estimate expression levels from a known transcript model. The min-isoform-fraction parameter of Cufflinks was set to 0.0 in order to recover very lowly expressed transcripts. The sequence alignment files generated from Bowtie were used for estimating transcript expression levels by both IsoformEx and Cufflinks, thus eliminating any plausible bias that could arise from different alignments files.

Table 1 shows estimation errors and correlation coefficients between estimated expression levels (v_est_) obtained by IsoformEx and true expression levels (v_true_) based on the simulated mRNA-Seq data. A true proportion vector was prepared by true expression values divided by the sum of true expression values (*i.e*., p_true = _v_true_/∑v_true_). A proportion vector of the estimated value was prepared by the estimated expression values divided by total estimated expression values. The error was defined as the mean value of absolute difference values between two proportion vectors, *i.e*., ∑_*i *_= _1_|p_true _(*i*) - p_est _(*i*)|/n, where p_true _(*i*) is the *i-*th element of the proportion vector of true expression values, p_est _(*i*) is the *i-*th element of the proportion vector of estimated expression values, and n is the number of estimated transcripts. IsoformEx showed the low error and high correlation coefficient for the simulated data. For more rigorous evaluation, we excluded trivial cases of single transcript genes (or transcript blocks with only one transcript), since the main goal of IsoformEx is to obtain estimates at isoform-level. We also excluded transcripts in mitochondrial DNA. When both expressed and non-expressed transcripts (v_true _≥ 0) were considered for the analysis, IsoformEx showed lower error and higher correlation coefficient in comparison to RSEM (v1.1.8) and Cufflinks (v0.9.3) with default/changed parameters (see Table 1 and Additional File 1 Tables S3-S4). By removing very lowly expressed (v_est _≤ 0.01) and the unexpressed transcripts, the error was increased because the absolute errors of other transcripts with v_est _> 0.01 were generally larger than those of unexpressed transcripts or very lowly expressed transcripts. We did not observe any significant variation of correlation coefficients by removing very lowly expressed transcripts. Overall, the results showed that the performance of IsoformEx was better than the other methods for estimating transcript expression levels, with the least error and the highest correlation coefficient.

**Table 1:** Performance Comparison on the simulated mRNA-Seq data

Algorithms	Condition	IsoformEx	**RSEM **[21] (v1.1.8) with default parameters	**Cufflinks **[20] (v0.9.3) (--min-isoform-fraction 0)
The number of estimated transcripts (n)	v_est _≥ 0	55416	55441	55441
	
	v_est _> 0.01	35064	25967	25839

	v_est _≥ 0	8.41 × 10^-6^	1.02 × 10^-5^	9.23 × 10^-6^
	
	v_est _> 0.01	1.32 × 10^-5^	2.15 × 10^-5^	1.96 × 10^-5^

r = Corr(v_true _, v_est _)	v_est _≥ 0	0.921	0.839	0.913
	
	v_est _> 0.01	0.920	0.838	0.912

Estimation error and correlation coefficient between estimated expression levels (v_est_) and known true expression levels (v_true_) for our simulated dataset when all estimated transcripts (v_est _≥ 0) were considered or some expressed transcripts (v_est _> 0.01) were only considered. p_true_ (*i*) denotes the *i-*th element of the proportion vector of true expression values (p_true_ = v_true_/∑ v_true_), and p_est_ (*i*) denotes the *i-*th element of the proportion vector of estimated expression values (p_est_ = v_est_/∑ v_est_). The error was defined as the mean value of absolute difference between the true proportion vector and the proportion vector of the estimated values.

### Performance of IsoformEx on real mRNA-Seq data and qRT-PCR experiments

To validate the estimation of transcript isoforms from the real mRNA-Seq data of MCF-7 and HME cell lines [5], we performed quantitative RT-PCR (qRT-PCR) experiments on the same cell lines. We obtained qRT-PCR measurements for eight transcripts in four separate genome loci, such that each genomic locus contains two overlapping transcripts. For each of the four genomic loci, we calculated the expression ratio of one transcript over another in each cell line (MCF-7 and HME). Similarly, the expression ratios for each transcript in MCF-7 relative to HME cell line were also obtained (see Table 2). The changes of expression (up/down) of transcripts between cell lines and within cell lines, determined by qRT-PCR measurements, are in good agreement with IsoformEx estimates obtained by analyzing the mRNA-Seq data.

**Table 2:** qRT-PCR validation in human breast cell lines for IsoformEx

		qRT-PCR Poly(A) RNA			IsoformEx			**Cufflinks **[20] (v0.9.3) (--min-isoform-fraction 0)		
**Symbol**	**TranscriptID**	**HME**	**MCF-7**	**FC**_**b**_	**HME**	**MCF-7**	**FC**_**b**_	**HME**	**MCF-7**	**FC**_**b**_

*TRAP1*	uc002cvt.2	234.7	302.3	0.4	27.4	43.9	0.7	22.3	33.2	0.6

*TRAP1*	uc002cvs.1	423.5	595.4	0.5	40.9	56.0	0.5	0.8	1.6	1.0

	FC_w_	0.9	1.0		0.6	0.4		-4.8^†^	-4.4^†^	

*ZNF581*	uc002qlq.1	621.8	755.9	0.3	8.3	23.5	1.5	8.1	20.0	1.3

*ZNF580*	uc002qlp.1	277.9	381.3	0.5	7.7	14.4	0.9	2.2	5.3	1.3

	FC_w_	-1.2	-1.0		-0.1	-0.7		-1.9	-1.9	

*WISP2*	uc002xmn.1	10.7	530.0	5.6	1.8	14.7	3.0	0.0	12.0	6.9^‡^

*WISP2*	uc002xmo.1	8.1	189.6	4.5	0.5	8.4	4.2	0.0	4.2	5.4^‡^

	FC_w_	-0.4	-1.5		-2.0	-0.8		0.0^‡^	-1.5	

*HIST1H2BD*	uc003ngr.1	12.4	317.8	4.7	0.7	6.9	3.3	4.3	55.9	3.7

*HIST1H2BD*	uc003ngs.1	538.2	19207.9	5.2	10.9	136.3	3.6	0.9	16.2	4.1

	FC_w_	5.4	5.9		4.0	4.3		-2.2^†^	-1.8^†^	

FC_w_: log_2_(a/b), where a and b are the second/first transcript expression within the same cell line. FC_b_: log_2_(a/b), where a and b are the expression values of a transcript in MCF-7 and HME cell line, respectively. ^†^Fold change directions of Cufflinks estimations were erroneously flipped. ^‡^When b = 0, the fold change was computed from log_2_((a+0.1)/(b+0.1)).

**Figure 3:**
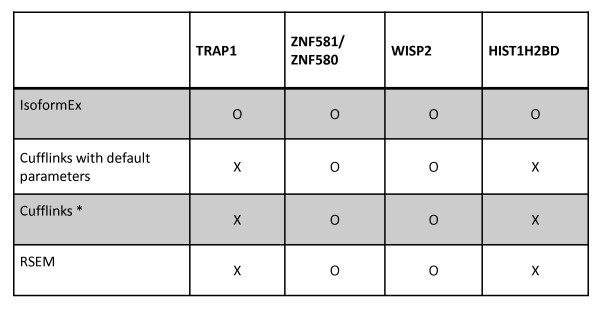
**Agreement between estimated RPKM values and qRT-PCR validation for four transcript blocks in breast cell lines**. We selected four transcript blocks and two transcripts for each transcript block. We compared qRT-PCR measurements and transcript expression levels estimated by several methods in the MCF7 cell line. When the corresponding method could correctly predict the direction of fold change within the HME cell line, 'o' mark is used. Otherwise, 'x' mark is used. Cufflinks*: the min-isoform-fraction parameter of Cufflinks was set to 0.0 in order to recover very low expressed transcripts. IsoformEx and Cufflinks* used the same Bowtie output files.

The correlation coefficients between qRT-PCR measurements and values estimated with IsoformEx, Cufflinks, and RSEM were 0.88, 0.08, and 0.05, respectively. The low correlation of Cufflinks and RSEM was due to up/down flips of fold changes (FC_w_) in transcript expression for *TRAP1 *block and *HIST1H2BD *block (see Figure 3). The different estimations for *HIST1H2BD *block was confirmed by custom wiggle track of mapped tags on the UCSC genome browser (see Additional File 1 Figure S2). By applying higher weight to the discriminative exon slice of uc003ngr.1 transcript and using the lower number of tags that mapped to the discriminative exon slice than the number of tags that map to the rest of the transcript region, IsoformEx estimated the expression of uc003ngr.1 as lower than that of the other transcript (uc00ngs.1). Other programs incorrectly estimated that uc003ngs.1 was expressed at lower level than uc00ngr.1 since they did not apply additional weight to the discriminative exon slice. For fair comparison, we used the same Bowtie output files for IsoformEx and Cufflinks in Table 2. We also tested Cufflinks with default parameters. Table 3 shows the agreement between qRT-PCR results and estimations obtained from RSEM and Cufflinks with default parameters. They generated two up/down flips of FC_w _(fold change within cell line). Cufflinks did not show any substantial changes in performance with different values of the min-isoform-fraction parameter. IsoformEx showed the best agreement with qRT-PCR measurements without any fold change direction flips.

**Table 3:** qRT-PCR validation in human breast cell lines for other methods

		qRT-PCR poly(A) RNA			**RSEM **[21] (v1.1.8) with default parameters			**Cufflinks **[20] (v0.9.3) with default parameters		
**Symbol**	**TranscriptID**	**HME**	**MCF-7**	**FC**_**b**_	**HME**	**MCF-7**	**FC**_**b**_	**HME**	**MCF-7**	**FC**_**b**_

*TRAP1*	uc002cvt.2	234.7	302.3	0.4	1378.0	1782.1	0.4	22.3	33.2	0.6

*TRAP1*	uc002cvs.1	423.5	595.4	0.5	47.0	82.0	0.8	0.8	1.7	1.1

	FC_w_	0.9	1.0		-4.9^†^	-4.4^†^		-4.8^†^	-4.3^†^	

*ZNF581*	uc002qlq.1	621.8	755.9	0.3	358.9	687.6	0.9	8.6	20.0	1.2

*ZNF580*	uc002qlp.1	277.9	381.3	0.5	148.1	245.0	0.7	2.3	5.4	1.2

	FC_w_	-1.2	-1.0		-1.3	-1.5		-1.9	-1.9	

*WISP2*	uc002xmn.1	10.7	530.0	5.6	10.0	435.0	5.4	0.0	11.8	6.9^‡^

*WISP2*	uc002xmo.1	8.1	189.6	4.5	9.7	250.9	4.7	0.0	4.2	5.4^‡^

	FC_w_	-0.4	-1.5		0.0	-0.8		0.0^‡^	-1.5	

*HIST1H2BD*	uc003ngr.1	12.4	317.8	4.7	77.5	566.1	2.9	4.4	55.9	3.7

*HIST1H2BD*	uc003ngs.1	538.2	19207.9	5.2	21.7	499.0	4.5	0.9	16.2	4.2

	FC_w_	5.4	5.9		-1.8^†^	-0.2^†^		-2.4^†^	-1.8^†^	

FC_w_: log_2_(a/b), where a and b are the second/first transcript expression within the same cell line. FC_b_: log_2_(a/b), where a and b are the expression values of a transcript in MCF-7 and HME cell line, respectively. ^†^Fold change directions of estimations were erroneously flipped. ^‡^When b = 0, the fold change was computed from log_2_((a+0.1)/(b+0.1)).

We also compared the execution times of IsoformEx and other programs for reading the alignment files generated by Bowtie and estimating transcript isoform levels from mRNA-Seq data for MCF-7 cell line. Each execution time was measured in the same computer (two Intel^® ^Xeon^® ^CPUs X5460 (12 M Cache, 3.16 GHz, 1333 MHz FSB, 4 cores), 36 GB memory). Cufflinks only accepts SAM file format [27] as an input file format, whereas IsoformEx can handle Bowtie output file format as well as SAM file format. Cufflinks (v0.9.3, machine code) took about 40 minutes with a SAM file for estimating transcript expression levels. RSEM (v1.1.8, machine code) took about five hours including mapping short reads to transcriptome and estimating transcript expression levels. IsoformEx (Matlab code) took about 40 minutes with two Bowtie output files in order to estimate transcript expression levels of all valid transcripts and gene expression levels. Although this is not rigorous comparison, the result shows the computational efficiency of IsoformEx.

## Discussion

In the current implementation, IsoformEx assumes a transcript model is given and the transcript model has most dominantly expressed isoforms. While IsoformEx addresses the problem of accurately estimating the isoform-level expression values, by assigning reads to a known set of splice-variants, Cufflinks attempts to simultaneously discover novel isoforms and estimate their expression values. Using user defined transcript model having novel isoform candidates would be a possible way for improving estimation accuracy. IsoformEx can handle overlapping genes as well as overlapping exons in transcript model for more accurate estimation of gene/transcript expression from mRNA-Seq data. It converts the transcript abundance estimation problem to a constrained optimization problem in order to take advantage of non-negativity in transcript expression levels. It uses the non-negative least squares algorithm which is well established optimization method based on mathematical/numerical analysis. It is numerically stable and computationally efficient. The non-negative least squares framework is simple and mathematically well defined so that additional biological knowledge can be incorporated in the framework through a weighting scheme. For example, the number of short reads in a smaller exon slice tends to be small due to the limitation of mRNA-Seq coverage. IsoformEx uses a weighting scheme for considering the small exon effect. Although we suggested a reasonable weighting scheme, it would be worthwhile to study other weighting schemes. IsoformEx can be applied to mRNA-Seq data analysis not only to estimate expression levels of transcript isoforms, but also to estimate more accurate gene expression levels when different genes overlap. Executable machine codes are available at http://bioinformatics.wistar.upenn.edu/isoformex.

## Conclusion

We developed IsoformEx algorithm to estimate transcript-level expression values from mRNA-Seq sequence reads that were mapped to the genome. It can be applied not only to estimate isoform-level mRNA expression values, but also to estimate gene-level expressions when different genes overlap. Executable machine codes are available at http://bioinformatics.wistar.upenn.edu/isoformex.

## Methods

### Real mRNA-Seq data

We downloaded mRNA-Seq data for human breast cancer cell line MCF-7 (GEO sample GSM325487, SRA experiment SRX003923) and human breast normal cell line HME [5] (GEO sample GSM325485, SRA experiment SRX003921) from the Short Read Archive section of GEO at NCBI [28] (accession number GSE12946 (alternative isoform regulation in human tissue transcriptomes) on the platform GPL9052 (Illumina genome analyzer, Homo sapiens)). For qRT-PCR validations of our estimations of transcript expression levels from these data, we obtained the same cell lines for which mRNA-Seq experiments [5] were performed (MCF-7 cell line was purchased from ATCC (http://www.atcc.org/). HME cell line was obtained from Weinberg's lab). The raw FASTQ files have short-sequence reads of length 32 bp. The sequence reads that map to mitochondrial genome and ribosomal RNA sequences were filtered out. Reads were mapped to the human genome and splice junction database by Bowtie [26].

### IsoformEx based on non-negative least squares

The estimation method of IsoformEx is based on weighted non-negative least squares. Figure 1 shows the algorithm flow chart and an example to predict isoform expression levels from short read data using expression levels of exon slices. A transcript block consists of transcript isoforms of a gene and other transcripts overlapping them that belong to other genes. For each transcript block, IsoformEx collects slices of exons and computes values of RPKM [29] (reads per kilobase of exon per million mapped reads) of all exon slices and splice junctions. When we computed RPKM of splice junctions, the length of splice junction was fixed at 54. For splice junction mapping, the 32 bp tag should have at least five nucleotide matches from either side of splice junction.

Both constitutive exons and alternative exons were used for obtaining slices of exons. Thus, we build an exon structure matrix (A_*exon*_) of the transcript block, where A_*exon *_(*i,j*) *= *1 when the *j*-th slice is a part of the *i*-th transcript, otherwise A_*exon *_(*i,j*) *= *0. It also builds a splice junction structure matrix (*A_sj_*) of the transcript block, of which element *A_sj _*(*i,j*) = 1 when the *j*-th junction is a part of the *i*-th transcript, otherwise *A_sj _*(*i,j*) = 0. After combining the exon structure matrix and splice junction structure matrix, and combining a RPKM column vector (b_*exon*_) of exon slices and a RPKM vector (*b_sj_*) of splice junctions, we solve a weighted non-negative least squares problem for each transcript block,(1)

where A = [*A*_exon _A_sj_ ] is the combined exon structure matrix, *b *= [*b*_exon_;*b* {_sj_}] is the combined column vector having all RPKM values, *x *is the solution vector having transcript isoform expression levels, and *W *is the weight diagonal matrix whose diagonal elements are the corresponding weights of exon slice or splice junction. The observed short reads in an exon slice is actually the sum of short reads generated from different transcript isoforms having the exon slice. We identify the contribution of individual transcripts when exon slices are shared by multiple transcripts. But, sometimes, the number of reads of an exon slice is small just because the exon slice is short. A weighting scheme was used since the confidence level of the number of reads in smaller exon slice is lower. In addition, the total concentration of transcript isoforms should be zero or positive. Thus, we constructed a weighted non-negativity-constraint optimization problem. This problem can be solved by a non-negative least squares algorithm. After estimating expression levels of transcripts, we estimated an expression level for each gene by the sum of the expression levels of the individual transcript isoforms of the gene.

The non-negative least squares problem has a unique solution when *A *is full rank and the algorithm converges to the solution [30]. When *A *is rank-deficient, the code of *lsqnonneg *in Matlab (Matlab2009a, Natick, MA) solves the non-negative least squares problem with a linearly independent subset of the columns of *A^T^*and leaves all other values of *x *at zero. This is a particular solution to the minimization problem subject to non-negativity constraints. Here is the simplest example of rank-deficient *A *generated from four transcripts having three constitutive exons and two splice junctions:

The sum of the first and second rows of *A *is same as the sum of the third and fourth rows of *A*. The second row vector equals the third plus the fourth minus the first, *i.e*. A(2,:) = *A*(3,:) + *A*(4,:) - *A*(1,:). The maximal number of linearly independent columns of *A *is 3, i.e. rank(*A*) = 3. The rank of 4 × 5 matrix *A *is smaller than min(4, 5) = 4, so *A *is rank-deficient. Such non-identifiable cases were already discussed in the previous works [20,31]. If a larger transcript block has above four transcripts, the bigger structure matrix is also rank deficient and the problem does not have a unique solution. A more complicated example having four transcripts, four constitutive exons and four splice junctions follows:

The sum of the first and third rows of *A *is same as the sum of the second and fourth rows of *A*. The rank of *A *is 3, so *A *is rank-deficient. Although these examples contain only constitutive exons without any alternative exon, having only constitutive exons does not guarantee non-identifiability. The simple rule for detecting non-identifiability of a transcript block is testing the rank-deficiency of structure matrix *A *and convergence of a non-negative least squares problem. Although it is theoretically possible to have non-identifiable cases, the frequency of non-identifiable cases was lower than 2% of clusters in our estimation of transcript expression levels in MCF7 cell line, whereas the percentages of non-identifiable models under the Affymetrix Exon 1.0 ST array (four probes per targeting exons) and the Affymetrix Human Exon Junction array (HJAY) (eight probes per targeting exons or splice junctions) were 74% and 31% for 2256 alternative spliced human genes [31].

When the length of exon slice is smaller than the tag length of 32 bp, no reads can be detected. We excluded such small exon slices for accurate estimation. Even if the length of an exon slice is larger than the tag length, the smaller exon tends to have the smaller number of mapped reads. We defined a weight saturation curve to represent the confidence level of RPKM with respect to lengths of slices, *i.e*. w = 1-exp(-*x*/100), where *x *is the length of an exon slice (see Additional File 1 Figure S3). The exon slice length confidence level starts from 0 at length = 0, gradually increases to 1.0 from around length = 1000 bp. When length = 500 bp, the length confidence level is already reached to *w*≈0.99. If an exon slice is only used for a transcript in the block, the exon slice is called as a discriminative exon slice and is highly weighted if the exon slice length confidence is larger than 0.5. Thus, a discriminative exon slice whose length is very small was not highly weighted; instead, it is lowly weighted due to low slice length confidence. As for splice junctions, the junction length confidence was fixed (*w*≈0.42) since we used a fixed length of splice junction (54 bp) and the same saturation curve for the confidence level. Basically, RPKMs for splice junctions were lowly weighted, but discriminative splice junctions were highly weighted. The confidence level for each slice was multiplied to the corresponding column of the structure matrix *A *and the vector element of RPKMs so as to construct a weighted non-negative least squares problem. As for discriminative exon slices, the confidence level was increased by *w *= *w *+ *w*_e_, where *w*_e _= 10 is the additional weight for discriminative exon slices. As for discriminative splice junctions, the confidence level was increased by *w *= *w *+ *w*_s_, where *w*_s _= 2 is the addition weight for discriminative splice junctions. When a transcript has discriminative splice junctions as well as discriminative exon slices, the confidence levels of discriminative splice junctions were not increased so that expression level of the transcript can be determined by more reliable RPKM values of discriminative exon slices.

### qRT-PCR experiments

We performed qRT-PCR experiments on poly(A) purified RNA from MCF-7 and HME cell lines. Approximately 1 μg or 100 ng of poly(A) purified RNA was reverse transcribed (RT) to generate cDNA using Superscript II following DNAse I treatment according to manufacturer's instructions (Invitrogen Inc.). We designed primers that would uniquely amplify a single transcript isoform from the UCSC transcript database to perform quantitative PCR. The forward/reverse primer sequences and their genomic locations can be found in the Additional File 1 Table S5. Using the specific primers for eight distinct mRNA isoforms that are localized in four distinct gene loci we performed SYBR green based PCR on the reverse transcribed cDNA for absolute quantification of each mRNA isoform in the two cell lines.

## Authors' contributions

HK and RVD conceived the initial approach. HK designed and implemented the methods. HK, YB, and RG performed the analyses. SP performed validation experiments. HK wrote the first draft of the manuscript. All authors contributed to editing and writing of the manuscript. All authors read and approved the final manuscript.

## Supplementary Material

Additional file 1 **Supplementary Material**. Supplementary tables and figures. Click here for file
