# Supplementary material for: IsoformEx: isoform level gene expression estimation using weighted non-negative least squares from mRNA-Seq data
Source: BMC Bioinformatics. 2011 Jul 27;12:305. doi: 10.1186/1471-2105-12-305 (PMC3180389; doi:10.1186/1471-2105-12-305)
Supplement: Additional file 1 — Supplementary Material. Supplementary tables and figures. [file 1471-2105-12-305-S1.PDF]

Supplementary Material

IsoformEx: Isoform level gene expression estimation using weighted non-negative least squares from mRNA-Seq data

Hyunsoo Kim[1], Yingtao Bi[1], Sharmistha Pal[1], Ravi Gupta[1], Ramana V. Davuluri[1]

[1]Center for Systems and Computational Biology, The Wistar Institute, 3601 Spruce Street, Philadelphia, PA 19104-4268, USA

Supplementary Table 1 – The exon slices of *ZNF581*/*ZNF580* cluster. The size of the sixth exon slice (s6) is 19, which is smaller than tag size in mRNA-Seq. The usage of exon slices for each transcript is indicated by 1. For example, uc010etc is only using the seventh slice (s7).

ID	Gene	s1	s2	s3	s4	s5	s6	s7	s8
uc002qlm	ZNF580	1	0	0	1	0	0	0	0
uc002qln	ZNF581	1	0	0	0	0	1	1	1
uc002qlo	ZNF580	0	1	0	1	0	0	0	0
uc002qlp	ZNF580	0	0	1	1	0	0	0	0
uc002qlq	ZNF581	0	0	0	0	1	1	1	1
uc010etc	ZNF581	0	0	0	0	0	0	1	0
Slice Length		697	158	445	974	155	19	594	458

Supplementary Table 2 – The splice junctions of *ZNF581*/*ZNF580* cluster. The usage of splice junctions for each transcript is indicated by 1. For example, uc002qlm has only one splice junction (s1-s4) between two exon slices (s1 and s4).

ID	Gene	s1-s4	s1-s6	s2-s4	s5-s6
uc002qlm	ZNF580	1	0	0	0
uc002qln	ZNF581	0	1	0	0
uc002qlo	ZNF580	0	0	1	0
uc002qlp	ZNF580	0	0	0	0
uc002qlq	ZNF581	0	0	0	1
uc010etc	ZNF581	0	0	0	0

Supplementary Table 3 – Performance comparison on the simulated mRNA-Seq data for IsoformEx and Cufflinks [1] with default parameters. Estimation error and correlation coefficient between estimated expression levels ( ) and known true expression levels ( ) for our simulated dataset when all estimated transcripts ( ) were considered or some expressed transcripts ( ) were only considered. denotes the *i*-th element of the proportion vector of true expression values ( ), and denotes the *i*-th element of the proportion vector of estimated expression values ( ). The error was defined as the mean value of absolute difference between the true proportion vector and the proportion vector of the estimated values.

Algorithms	Condition	IsoformEx	Cufflinks (v0.9.3) with default parameters	Cufflinks (v0.8.2) with default parameters
The number of estimated transcripts ( )		55416	55441	20020
		35064	25803	19999
Error=		8.41×10^{-6}	9.09×10^{-6}	1.68×10^{-5}
		1.32×10^{-5}	1.94×10^{-5}	1.68×10^{-5}
=Corr(,)		0.921	0.917	0.876
		0.920	0.917	0.876

Supplementary Table 4 – Performance comparison on the simulated mRNA-Seq data for IsoformEx and RSEM [2] with additional parameters in addition to common parameters (--phred64-quals --seed-length 30). Estimation error and correlation coefficient between estimated expression levels ( ) and known true expression levels ( ) for our simulated dataset when all estimated transcripts ( ) were considered or some expressed transcripts ( ) were only considered. denotes the *i*-th element of the proportion vector of true expression values ( ), and denotes the *i*-th element of the proportion vector of estimated expression values ( ). The error was defined as the mean value of absolute difference between the true proportion vector and the proportion vector of the estimated values.

Algorithms	Condition	IsoformEx	RSEM (v1.1.8) with additional parameters (--bowtie-m 10)	RSEM (v1.1.8) with additional parameters (--fragment-length-mean 200)
The number of estimated transcripts ( )		55416	55441	55441
		35064	25332	26981
Error=		8.41×10^{-6}	1.06×10^{-5}	1.09×10^{-5}
		1.32×10^{-5}	2.26×10^{-5}	2.21×10^{-5}
=Corr(,)		0.921	0.830	0.825
		0.920	0.831	0.826

Supplementary Table 5 – qRT-PCR primers and genomic locations obtained from the UCSC in-silico PCR [3].

Symbol	TranscriptID	Forward primer sequence	Reverse primer sequence	Genomic locations in Hg18
TRAP1	uc002cvt.2	GCCATGTCGTACTCCCAGA	CCTTCCCATCGTGTACGG	chr16:3707506-3707565
TRAP1	uc002cvs.1	GAGAGCAGACACTCCCAACA	ATGGGGTGGAAAAGTACTCG	chr16:3667702-3667796
ZNF581	uc002qlq.1	TCCCTTCGGCTTCTCTCTT	AAGGGGACCTCTGGGTGT	chr19:60846813-60846876
ZNF580	uc002qlp.1	GGTGGGTTGAGAGGAGAAAA	CAACTGAGCTCTGCAAAACC	chr19:60845359-60845482
WISP2	uc002xmn.1	TTAGGAGACCTTGGGTCAGC	GTGAAGCCCTATTCCAGACC	chr20:42776935-42777038
WISP2	uc002xmo.1	TTCCAGCTGAACTTGGTGTC	GTTGGCAATGATTTGGACAG	chr20:42782295-42782421
HIST1H2BD	uc003ngr.1	GCATCTTTACACCTAATCCCAAA	GAAAACATGCGTGGCTCTTA	chr6:26266771-26266821
HIST1H2BD	uc003ngs.1	GCCTGAAAATGACTGTGTGG	CAGCAAACCAGGATGAGTTG	chr6:26279268-26279321

Supplementary Figure 1 – A flowchart of a simple example for describing basic logic of expression estimation with RPKM values of exon slices of *ZNF580* and *ZNF581* (see Figure 2). The α(·) is the RPKM of an exon slice. For example, the expression level of the fifth exon slice s5 is α(s5). The (·) is the RPKM of a transcript. For example, the expression level of uc002qlq is (uc002qlq). The approximated RPKM values of exon slices can be found in Figure 2. This toy example is only designed for explaining the importance of non-negativity concept in estimation. Actual estimation is much more complex than this.

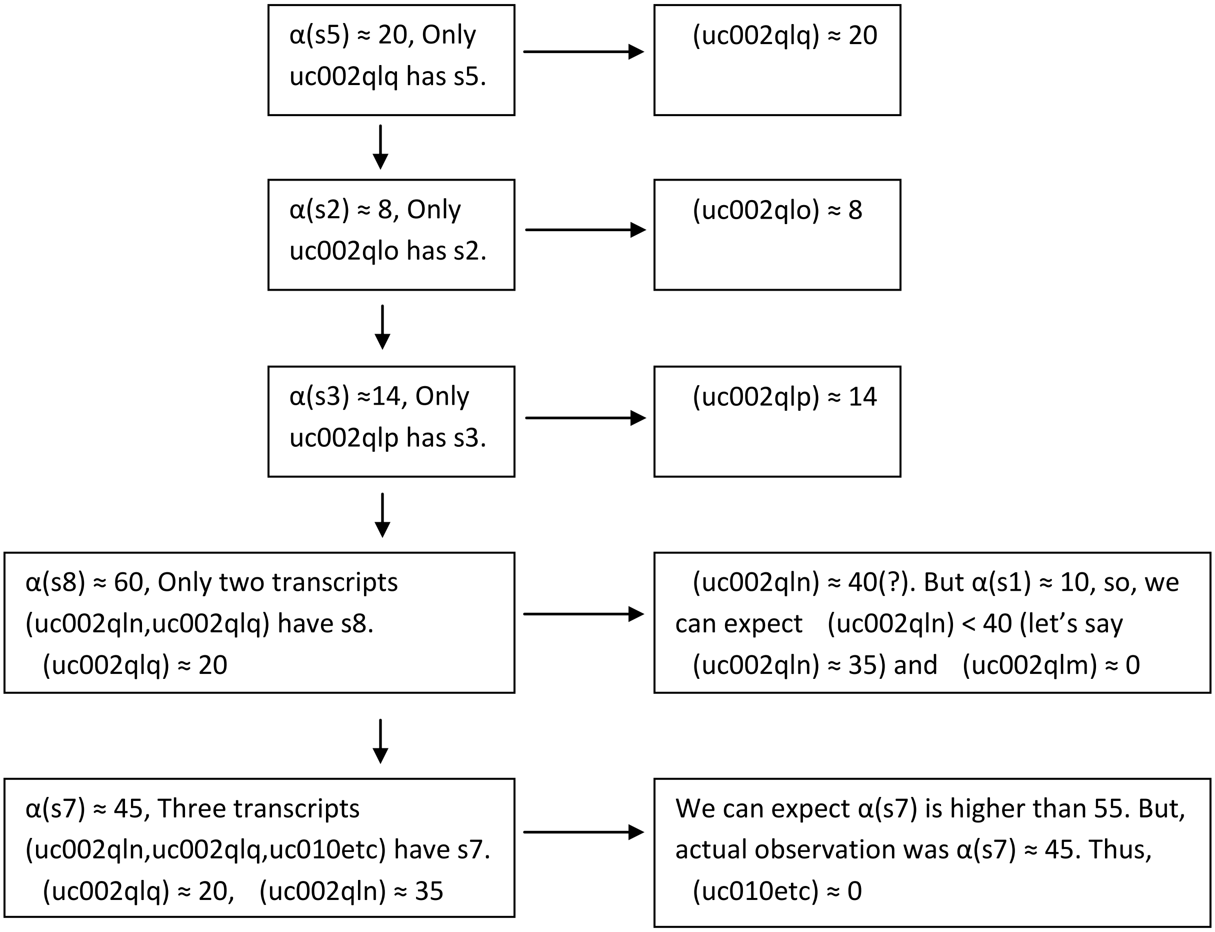

Supplementary Figure 2 – Custom wiggle track of mapped tags on the UCSC genome browser [3] for *HIST1H2BD* in the HME cell line [4]. There are two transcripts for *HIST1H2BD, i.e.* uc003ngr.1 (upper transcript in this figure) and uc003ngs.1 (lower transcript). (a) Wiggle track around HIST1H2BD gene, (b) Wiggle track around the 3' UTR region of uc003ngr.1. The number of tags inside the discriminative exon slice of uc003ngr.1 is very small. The discriminative exon slice of uc003ngr.1 is a part of 3' UTR region of uc003ngr.1 (upper transcript). From this observation, we can expect that uc003ngr.1 is expressed less than uc003ngs.1. IsoformEx also estimated this tendency, which was confirmed by qRT-PCR (see Table 2).

(a)

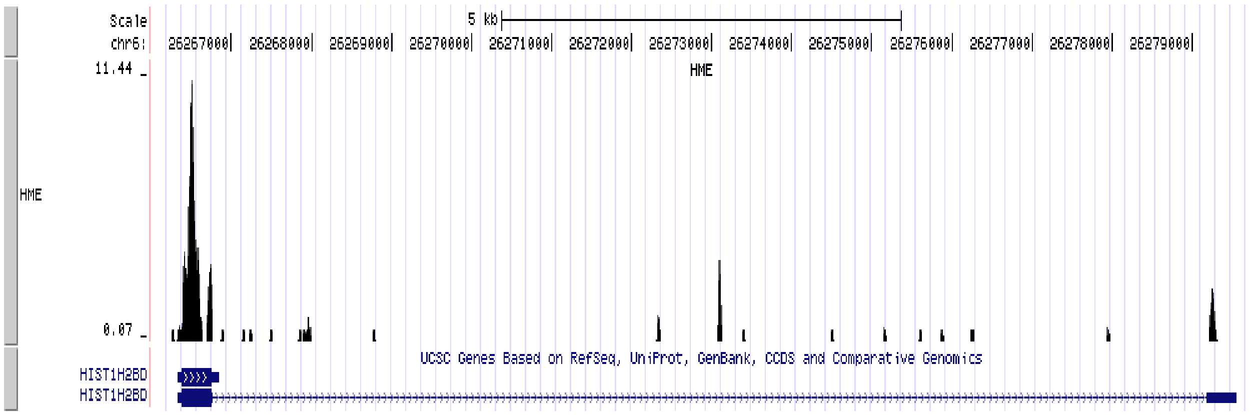

(b)

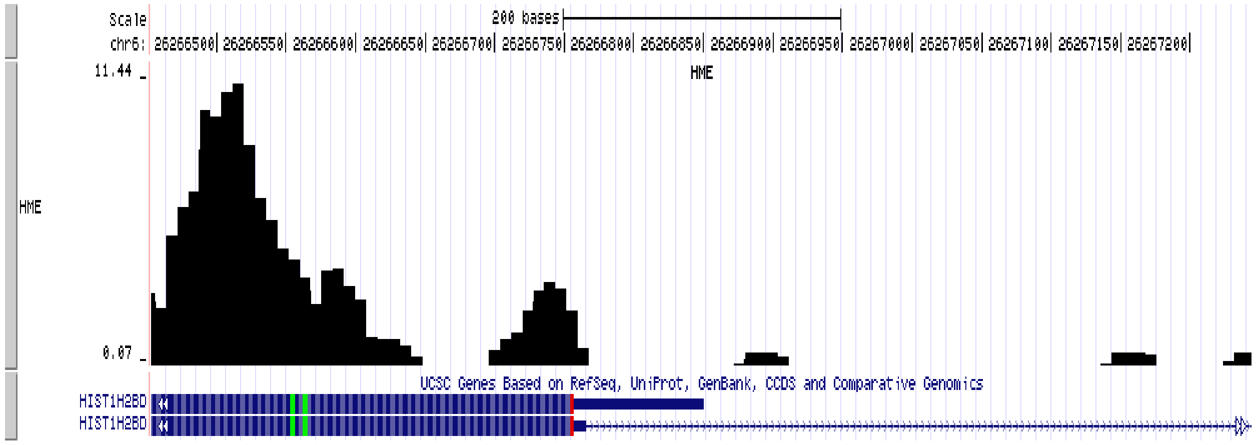

Supplementary Figure 3 – The weight saturation curve to present the confidence level of RPKM with respect to lengths of exon slices, i.e. w=1-exp(-x/100), where x is the length of exon slice. When x=70 (bp), the confidence is w≈0.5. When x=500 (bp), the confidence is already reached w≈0.99. As for splice junction, we fixed x=54 (bp), so the fixed confidence level of RPKM of splice junctions is w≈0.42.

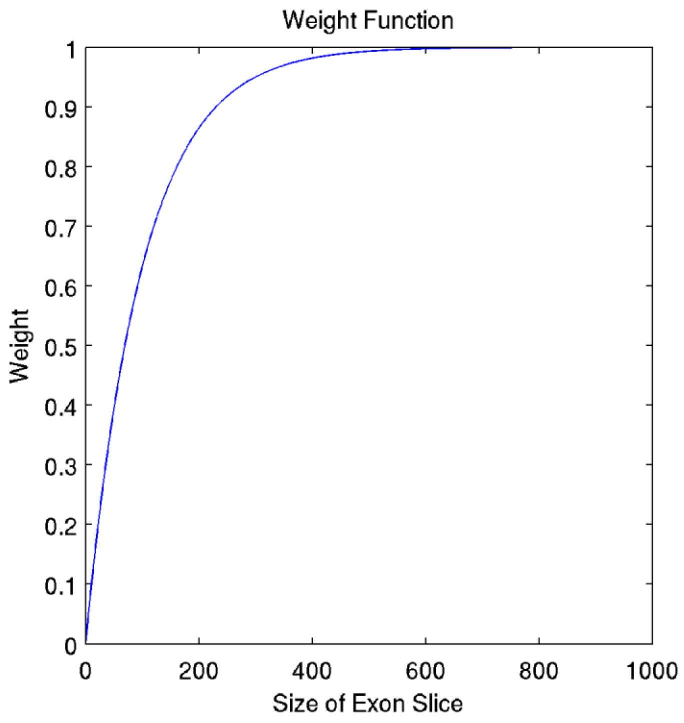

References

1. Trapnell C, Williams BA, Pertea G, Mortazavi A, Kwan G, van Baren MJ, Salzberg SL, Wold BJ, Pachter L: **Transcript assembly and quantification by RNA-Seq reveals unannotated transcripts and isoform switching during cell differentiation.** *Nat Biotechnol*, **28:**511-515.
2. Li B, Ruotti V, Stewart RM, Thomson JA, Dewey CN: **RNA-Seq gene expression estimation with read mapping uncertainty.** *Bioinformatics*, **26:**493-500.
3. Rhead B, Karolchik D, Kuhn RM, Hinrichs AS, Zweig AS, Fujita PA, Diekhans M, Smith KE, Rosenbloom KR, Raney BJ, et al: **The UCSC Genome Browser database: update 2010.** *Nucleic Acids Res*, **38:**D613-619.
4. Wang ET, Sandberg R, Luo S, Khrebtukova I, Zhang L, Mayr C, Kingsmore SF, Schroth GP, Burge CB: **Alternative isoform regulation in human tissue transcriptomes.** *Nature* 2008, **456:**470-476.
